# Investigation of spillover effects of a sugar-sweetened beverage tax on beverage purchasing in a nearby, non-taxed area: A quasi-experimental, difference-in-differences analysis

**DOI:** 10.1371/journal.pone.0340577

**Published:** 2026-02-04

**Authors:** Leah R. Neff Warner, Melissa A. Knox, Amanda M. Fretts, Brian E. Saelens, Jessica C. Jones-Smith

**Affiliations:** 1 Department of Epidemiology, University of Washington, Seattle, Washington, United States of America; 2 Department of Economics, University of Washington, Seattle, Washington, United States of America; 3 Center for Child Health, Behavior, and Development, Seattle Children’s Research Institute, Seattle, Washington, United States of America; 4 Department of Pediatrics and Department of Psychiatry and Behavioral Sciences, School of Medicine, University of Washington, Seattle, Washington, United States of America; 5 Department of Health Systems and Population Health, University of Washington, Seattle, Washington, United States of America; 6 Department of Health, Behavior, and Society, Joe C. Wen School of Population and Public Health, University of California, Irvine, California, United States of America; Wuhan University, CHINA

## Abstract

Evidence suggests that sugar-sweetened beverage (SSB) taxes reduce SSB purchasing and improve health outcomes in the taxed area. The extent to which purchasing also changes in nearby communities due to tax signaling effects is unclear. The objective of this study was to assess whether the SSB tax in Seattle, Washington, USA, influenced SSB purchasing in nearby communities within the same media market. We used retail scanner data on weekly sales of 3,531 beverages from 127 retailers in King County excluding Seattle and its bordering area (KC), and 243 retailers in a matched comparison area outside the regional media market. Matching was done via Mahalanobis distance based on pre-tax, county-level demographic measures from the American Community Survey. We estimated linear difference-in-differences in mean volume sold of taxed and nontaxed beverages comparing two years before (2016−2017) and after tax implementation (2018−2019) adjusting for beverage-level fixed effects. We also estimated the difference-in-differences in Seattle versus a matched comparison to estimate the tax effect in Seattle as context for potential effects in KC. For taxed beverages, the mean difference-in-differences in volume sold in KC was 172 liters (95% CI: −1,396, 1,740; P = 0.83), reflecting a 1% change from pre-tax levels in KC for a given beverage. There was suggestive evidence of increased volume sold for taxed and nontaxed soda, and taxed multipack beverages in KC relative to the comparison area. In Seattle, the mean difference-in-differences in volume sold for taxed beverages was −3,628 liters (95% CI: −4,622, −2,634; P < 0.001), reflecting a 20% decline for a given beverage in association with the tax. We did not find evidence of spillover effects in the form of reduced volume sold of SSB in communities near but not bordering the tax in Seattle. Studies in other contexts are needed to investigate spillover on purchasing as well as possible explanations for observed increases in purchases of taxed and nontaxed soda.

## Introduction

Sugar-sweetened beverages (SSB) are the largest source of added sugars in the American diet [1–3], contributing significantly to the risk of obesity, diabetes, and cardiovascular diseases [4,5]. In response, more than 50 jurisdictions worldwide, including eight in the US, have enacted SSB taxes in the past decade to reduce consumption and improve public health [6]. The rationale for such taxes is supported by strong evidence showing reductions in SSB purchasing in the taxed areas and emerging signs of health impacts such as reduced weight gain and dental caries at the population level [7–9]. However, the effect of these taxes on self-reported SSB consumption has been inconsistent, with some studies showing modest reductions only among populations with high consumption levels or low incomes [9–11].

Extending this research, there is growing interest in understanding potential spillover effects—how these taxes influence purchasing and consumption in nearby, nontaxed areas. This topic is important for assessing the broader public health implications of SSB taxes. It is also important for understanding inconsistent outcomes from studies since nearby comparison areas may be more vulnerable to spillover effects than comparison areas farther away [11]. Spillovers can include positive public health impacts whereby residents in neighboring regions reduce SSB consumption in response to health risk signaling of SSB taxes. On the other hand, residents from the taxed area may shop in neighboring regions to avoid the tax, known as cross-border shopping, potentially undermining tax effects on SSB consumption and health [12].

In 2018, the City of Seattle, Washington, implemented a 1.75-cent-per-ounce tax on the distribution of SSB [13]. As with other SSB taxes in the US, extensive research on this tax showed substantial and sustained reductions in SSB purchasing in Seattle [14,15]. However, in a longitudinal cohort study of families with lower income in Seattle and nearby, non-bordering communities, self-reported SSB consumption decreased in both groups, but not differentially [16,17]. In the present study, we investigated the question of whether tax spillover effects may explain the findings in nearby, non-bordering communities around Seattle.

While there is evidence of some cross-border shopping in most SSB purchasing studies, the extent to which it occurs tends to vary by location, and it does not typically offset the net decreases in purchasing in the taxed areas [9,11]. This is well documented in the context of tobacco and alcohol taxation [18,19]. In the case of Seattle’s SSB tax, cross-border shopping was not detected in areas immediately surrounding the city border [14,20].

Research on spillovers due to health risk signaling is relatively limited. In the taxed area, a signaling effect has been shown to influence behavior more than price increases alone in several [21–25], but not all studies [26]. For spillover to happen, residents living in nearby areas may be exposed to signaling effects through a shared media market or to messaging while spending time in the taxed area. While this type of spillover is a methodological consideration when selecting comparison areas for tax evaluations [11], to our knowledge no studies have assessed SSB purchasing or consumption in nearby non-taxed areas beyond the immediate border areas prone to cross-border shopping.

### Objective

This study assessed whether there is evidence of spillover of Seattle’s SSB tax on SSB purchasing in nearby communities within the same media market. We examined changes in volume sold of SSB before and after implementation of the Seattle Sweetened Beverage Tax among retailers in King County, Washington, that were not in Seattle or directly bordering Seattle, compared to changes in a matched comparison area drawn from outside of the Seattle media market. We also compared changes in volume sold in Seattle, relative to a matched comparison area outside of the media market. While tax effects on purchasing in Seattle have been observed relative to the comparison area of Portland, Oregon [14,20], it was important to assess whether our study detected a tax effect in Seattle, using a different comparison area and statistical approach, to contextualize our assessment of spillover.

## Methods

### Study design

We used a longitudinal, quasi-experimental difference-in-differences (DD) design to compare changes in the volume of taxable beverages sold in areas exposed to the Seattle Sweetened Beverage Tax—the treated areas—to comparison areas unexposed to the tax from two years before and after tax implementation (2016–2017 vs. 2018–2019). The DD design is a common method for evaluating policy impacts when outcome data are available in treated and comparison groups before and after implementation. A key assumption of this design is that unobserved differences between treated and comparison groups are consistent over time, resulting in parallel trends in the outcome had the treated group not received the treatment [27]. The DD estimate is the observed difference in trends between the groups over time, representing the effect of the policy on the treated group, under the parallel trends assumption [27]. We selected the study period of two years before and two years after tax implementation so that we could estimate sustained behavior change in response to tax while limiting the impact of market and population changes that could weaken the parallel trends assumption over a longer period.

We defined and examined separately two treated areas: 1) King County, Washington, excluding Seattle (KC), which was not subject to the tax, but hypothesized to be subject to tax spillover effects, and 2) Seattle, Washington. We cannot directly estimate spillover effects by comparing KC and Seattle to one another because we need comparison areas to represent trends we would expect in the absence of any tax and spillover effects. Further, due to demographic differences between Seattle and KC populations, SSB purchasing may trend differently over time in each area. Therefore, we estimated the DD for each treated area relative to comparison areas matched on demographic measures. The comparison area for KC was the combination of Sacramento County, CA, and Oakland County, MI, and for Seattle, the combination of Dane County, WI, and Denver County, CO.

To select the comparison areas, we used a multi-step process that matched US counties with the treated areas on population-level variables thought to be associated with trends in SSB consumption [28], detailed in S1 File. First, we used Mahalanobis distance matching [29] to identify counties similar to each of the two treated areas based on pre-tax, county-level demographic covariates from the American Community Survey [30]. We opted for this method instead of propensity score matching because it was not reliable for identifying matches based on a single treated unit. From the list of county matches, we excluded counties with a history of passing or proposing a SSB tax in or near the county because media coverage about a proposed tax may influence SSB purchasing behavior [31–33] and we needed a comparison area unexposed to tax media coverage. We further narrowed the list by prioritizing counties with a similar pre-tax political climate. Finally, we examined annual estimates of per capita and total SSB volume sold in the pre-tax period and ruled out areas in which SSB purchasing did not trend similarly or have similar levels to the treated areas prior to tax implementation. For the final comparison areas, we combined the top two county matches for each treated area and took the average of the volume sold; each county contributed equal weight to the outcome. We used combined comparison areas to smooth out potential violations of parallel trends from any single county comparison area.

### Data source and sample

We used retail scanner data from the NielsenIQ Datasets from the Kilts Center for Marketing Data Center at The University of Chicago Booth School of Business [34]. The dataset includes point-of-sale information on weekly pricing, volume, and store characteristics from participating retailers across the US and has been used in numerous studies of tax impacts [11,14,35,36]. In our analysis dataset, each observation represented the weekly total volume sold for a beverage item from each store in the dataset. Beverage items were defined at the Universal Product Code (UPC) level and included information such as item description, brand, size, packaging, product group, and product module. The store types defined by NielsenIQ in the dataset were food, drug, mass merchandiser, and convenience stores from more than 90 participating retailers in the US. The sample of stores is estimated to cover more than half of the total volume of goods sold in grocery and drug stores and more than 30% in mass merchandiser stores in the US [34]. The volume sales coverage of convenience stores and across all stores in this particular study setting are unknown. We estimate that while our sample of Seattle stores includes approximately 18% of all food retail stores in Seattle [37], the volume sales coverage is higher given the greater representation of food and mass merchandiser stores in the NielsenIQ sample. The researchers accessed the data on October 15, 2022, and did not have access to information that could identify individuals or stores at any point during or after analysis.

We assigned treatment based on the store’s location using the first three digits of the ZIP code, which was the smallest geographic identifier available to us. Within King County, WA, we assigned stores with a ZIP code starting with 980 to the KC treated area and stores with a ZIP code starting with 981 to the Seattle treated area. Because only the first three digits were known, some stores that were assigned to the Seattle area may have been located outside the border of the city and tax. Likewise, the KC treated area may have included stores in King County that were located beyond the areas that we believed were most likely to experience tax spillover. Since the population density is higher in the areas we believe to be sensitive to spillover [30], we assume stores are distributed similarly and that our sample largely reflects purchases in the areas sensitive to spillover. Importantly, all stores in the city of Seattle in the dataset were assigned to the Seattle treated area. In addition, stores in the immediate border around Seattle where cross-border shopping would likely occur are also grouped in the Seattle treated area. We expected this type of treatment misclassification to bias estimates of the tax and spillover towards the null, meaning that estimated changes in beverage volume sold may be smaller in magnitude than in the absence of misclassification. Because previous studies found no evidence of cross-border shopping in bordering areas around Seattle [24,25], we expected this bias to have minimal impact on our estimates of changes in volume sold in Seattle and KC.

At the UPC level, we categorized beverages as taxed (i.e., would be subject to the tax) or nontaxed using product module and item descriptions in the dataset. The Seattle tax applies to beverages sweetened with caloric sweeteners, whereas beverages with artificial, non-caloric sweeteners (e.g., diet soda) are not taxed [13]. We classified beverages by type, with taxed beverages including soda, fruit drinks, bottled coffee and tea, energy drinks, and sports drinks. Nontaxed beverages included diet soda, 100% juice/diet fruit drinks, milk, bottled water (plain, flavored, or sparkling), diet/unsweetened bottled coffee, diet/unsweetened bottled tea, diet energy drinks, and diet sports drinks. We included nontaxed beverages because they serve as a comparator to taxed beverages as we do not expect the tax or spillover of the tax to reduce purchasing of nontaxed beverages in the same way. Further, we wanted to understand whether nontaxed beverage purchasing increased as consumers may switch to nontaxed beverages in response to the tax. We additionally categorized beverages by size, including single serving (≤1 liter), family size (>1 liter), and multipack (>1 beverages of any size per package sold) because tax pass-through and purchasing are known to vary by beverage size [14,15].

Due to ambiguous tax status, we excluded from our sample: self-serve and fountain drink purchases, beverages in powder or syrup form, and beverages designated by NielsenIQ as mixers and additions to alcoholic beverages. We then excluded beverages for which we could not determine taxable status or beverage type after searching online for details, which accounted for 0.8–1.0% of volume sold in each of the treated and comparison areas. Finally, we omitted observations in the three months preceding the tax and in the first month after the tax due to potential effects of tax anticipation and implementation delays, as was suggested in previous studies of beverage purchasing and Seattle’s tax [14,20].

To limit bias due to differences in the number of stores participating in the dataset over time and between the treated and comparison areas, we used a balanced sample of stores. This means we excluded stores that appeared only once in the dataset in either the pre- or post-tax period. We also restricted our sample to balanced UPCs, allowing us to compare changes in volume sold within the same beverages over time. We excluded UPCs that appeared only once in the pre- or post-tax periods, which amounted to excluding 5.5% of the total volume in our dataset for KC, 4.2% for the KC comparison area, 6.0% for Seattle, and 5.3% for the Seattle comparison area.

### Statistical analysis

**Fig 1:**
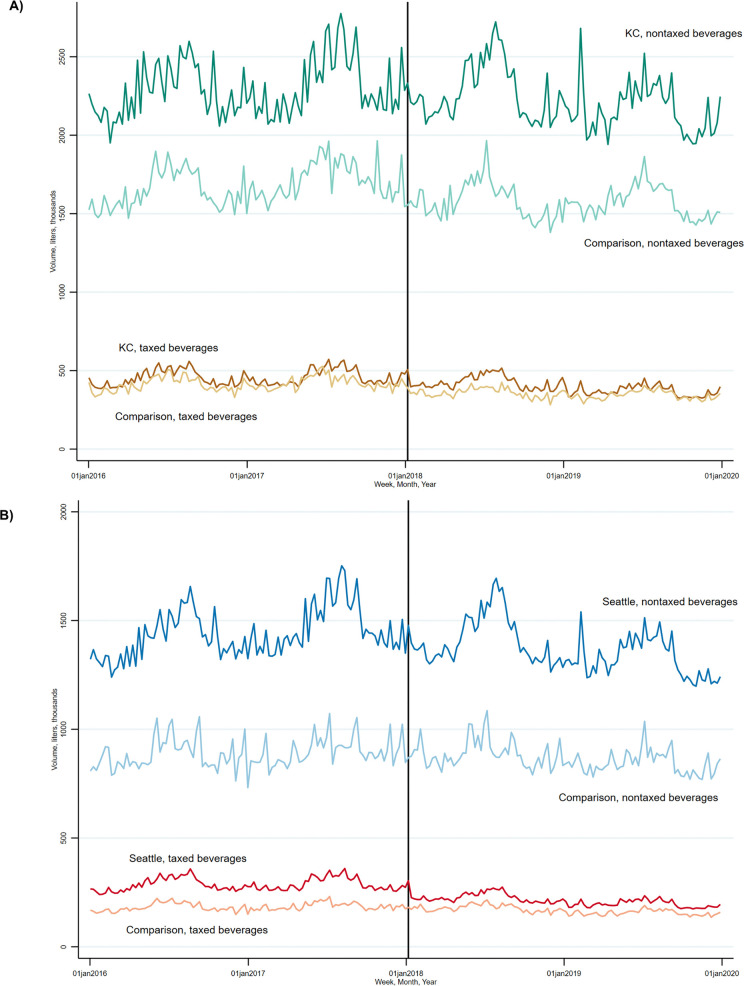
Weekly volume sold (liters) of beverages before and after implementation of Seattle’s Sweetened Beverage Tax, 2016-2019. The total weekly volume sold (liters) by taxable status in the balanced sample of stores and beverages in the two years before and two years after implementation of Seattle’s Sweetened Beverage Tax. **(A)** The treated area is King County excluding Seattle (KC) and comparison area is the combination of Sacramento, CA, and Oakland, MI, counties. **(B)** The treated area is Seattle and the comparison area is a combination of Dane, WI, and Denver, CO, counties.

We aggregated the total volume sold for beverages each in the pre-tax and post-tax periods by multiplying the UPC unit size in liters by units sold. To assess the degree to which trends in volume sold were evolving in parallel prior to policy implementation, we plotted mean weekly volume sold for beverages subject to the tax in the treated and comparison areas (Fig 1) and performed event study analyses [38] in the pre-tax period (S1 Fig). The event study analyses estimated the difference in mean volume sold for a typical beverage comparing the volume sold in each of the 24 months prior to tax implementation to the reference month of September 2017 (month −4) in each treated area relative to its comparison area. We selected this reference to mitigate anticipation effects immediately preceding tax implementation. An estimate of zero for a given month suggests the change relative to the reference month was not different between treated and comparison areas. In our analysis of each treated and comparison area pair, most monthly estimates hovered around zero, and those that differed from zero did not indicate a pattern of systematically diverging trends over time, for example, that the results were systematically lower and decreasing for the treatment versus comparison areas. Results supported the parallel trends assumption in that they did not provide evidence of systematically and meaningfully different pre-tax trends between treated and comparison areas.

For the primary analysis, we estimated the DD in the volume sold of taxed beverages from the pre-tax to post-tax periods in KC and the comparison area, and separately, in Seattle and the comparison area. We additionally estimated the DD for nontaxed beverages and for each beverage type and size category among taxed and nontaxed beverages. We fit linear DD regression models with UPC fixed effects and robust standard errors clustered at the UPC level. The DD, or tax effect estimate, reflects the average amount by which volume sold changed in the two years post-tax compared to two years pre-tax for a typical beverage in the treated area above and beyond changes in the comparison area. Fixed effects adjust for time-invariant, unobserved factors at the UPC level that may otherwise bias the tax effect estimate. The statistical model is described in S1 File.

In a secondary analysis, we estimated the DD in the first year post-tax because the tax may have influenced purchasing differently in the short term. We balanced the sample on stores and UPCs present one year before and after the tax, and applied the same exclusion criteria and fixed effects model above. In a separate analysis, we fit a pooled ordinary least squares regression model to estimate DDs using an unbalanced sample of UPCs over the two years before and two years after the tax. This added back to our sample beverages with purchases reported in only one of the pre- or post-tax periods. We estimated the DDs for the same categories of beverages described above, with covariate adjustment for beverage type and/or size. Instead of estimating within-UPC changes in the primary balanced sample, this analysis estimated the average change per UPC across all beverages in the context of compositional shifts in product availability over time. Estimates may take on more bias since we cannot adjust for unobserved UPC-specific characteristics. Further, the number of UPCs in each area and each timepoint differ and therefore affect the outcome of mean volume sold per UPC.

Additionally, we fit a linear regression model to estimate the triple difference (DDD) in volume sold—the difference between the KC DD and Seattle DDs—each for taxed and nontaxed beverages. This represented the amount by which the change in volume in Seattle that was attributed to the tax was different from any change in volume in KC that was attributed to spillover of the tax [39]. We used this model to test whether the DD in Seattle differed from the DD in KC; if both DDs were negative and not statistically significantly different from one another, this suggests a similar effect of the tax in Seattle and KC. We estimated the triple difference within each beverage type and size to examine differences in potential tax effects by beverage characteristics. We used the primary, balanced samples of UPCs and adjusted for UPC-level fixed effects and clustered, robust standard errors. The statistical model is described in the S1 File. A key assumption of a DDD analysis is that the relative differences between the pairs of treated and comparison areas trend similarly over time [39]; we extend our assumption of parallel trends in the primary DD models to this case.

Finally, we assessed the robustness of our findings to different model specifications and sample inclusion criteria. First, we explored whether results from the primary analysis differed substantively when using fixed effects for store-type-specific UPCs instead of UPCs from any store. For example, for this specification, we considered a UPC sold at a drug store to be a unique observation from the UPC sold at a mass merchandiser store. We explored this because there is evidence of differential pass-through of the tax across store types [15]. This specification allowed changes in volume sold for a beverage product to vary by store type, which could impact the average estimate of the tax effect for a given beverage. Then, we examined whether results were materially different when using a more restrictive definition of a unique UPC. In this specification, a beverage that changed its product size, brand, or description from one year to another was considered a separate beverage from its previous version and was thus less likely to be retained in the balanced sample. Results from both analyses were not materially different from those of the primary analysis.

For all analyses in this study, we set α to 0.05. Due to known limitations of using the P value to distinguish meaningful results, we did not correct for multiple comparisons and instead considered the magnitude of DD estimates, 95% confidence intervals, and P values altogether to guide interpretation of results [40]. All analyses were performed using Stata version 17.0 (College Station, TX).

## Results

The analysis samples included 1,541 UPCs from 127 stores in KC, 1,990 UPCs from 243 stores from the KC comparison area, 1,439 UPCs from 87 stores in Seattle, and 2,135 UPCs from 115 stores from the Seattle comparison area between the years 2016 and 2019. Tables 1 and 2 present mean volume sold for a typical UPC over time, by taxed status, beverage type, and size in each analysis sample. Fig 1 shows trends in weekly volume sold by taxed status for each analysis sample.

**Table 1:** Sample of beverages and mean volume sold (liters) by taxable status, type, and size in King County excluding Seattle (KC) and its combined comparison area, 2016-2019.

	KC (N stores = 127)					Comparison (N stores = 243)				
	N, UPCs	Pre-tax		Post-tax		N, UPCs	Pre-tax		Post-tax	
		Mean Volume, L	95% CI	Mean Volume, L	95% CI		Mean Volume, L	95% CI	Mean Volume, L	95% CI
**Taxed Beverages**										
Overall	1,541	27,324	(23,860, 30,788)	25,800	(22,403, 29,197)	1,990	19,291	(16,299, 22,283)	17,595	(14,916, 20,274)
*Beverage Type*										
Soda	614	30,604	(23,609, 37,598)	31,569	(24,471, 38,666)	817	23,348	(16,781, 29,915)	22,089	(16,249, 27,930)
Fruit Drinks	393	24,290	(19,023, 29,556)	18,616	(14,703, 22,529)	592	15,373	(12,272, 18,475)	12,097	(9,649, 14,546)
Bottled Coffee	67	12,356	(7,417, 17,295)	12,846	(7,297, 18,396)	77	5,527	(3,398, 7,656)	5,690	(3,330, 8,050)
Bottled Tea	228	15,917	(11,166, 20,668)	14,658	(9,935, 19,381)	246	12,618	(8,640, 16,596)	11,652	(7,540, 15,764)
Energy Drinks	79	24,970	(14,436, 35,505)	27,175	(16,119, 38,231)	81	11,676	(5,663, 17,689)	12,410	(5,818, 19,003)
Sports Drinks	95	54,260	(36,757, 71,762)	46,473	(29,280, 63,665)	106	41,324	(29,367, 53,281)	38,188	(26,239, 50,137)
*Beverage Size*										
Single Serving (≤ 1 L)	913	14,044	(11,536, 16,552)	13,857	(11,356, 16,357)	1,066	7,984	(6,408, 9,560)	7,975	(6,348, 9,601)
Multi-pack	342	34,175	(26,082, 42,268)	37,034	(28,742, 45,325)	466	23,659	(17,657, 29,662)	23,161	(17,994, 28,329)
Family Size (> 1 L)	286	61,527	(48,528, 74,525)	50,492	(37,880, 63,104)	458	41,164	(30,558, 51,769)	34,322	(24,846, 43,798)
**Nontaxed Beverages**										
Overall	3,594	58,869	(46,662, 71,077)	61,407	(48,708, 74,106)	4,313	35,796	(26,602, 44,989)	36,404	(26,707, 46,102)
*Beverage Type*										
Diet Soda	858	58,601	(46,398, 70,804)	66,047	(51,234, 80,860)	995	31,220	(24,030, 38,411)	31,803	(24,211, 39,396)
100% Juice/Diet Fruit Drinks	1,014	18,928	(15,984, 21,872)	15,119	(12,031, 18,206)	1,209	10,105	(8,359, 11,850)	7,662	(6,117, 9,206)
Milk	594	114,180	(57,572, 170,788)	115,814	(60,093, 171,534)	820	46,990	(16,980, 76,999)	44,788	(17,835, 71,741)
Bottled Coffee	72	6,562	(3,267, 9,857)	10,481	(5,685, 15,276)	75	2,902	(1,531, 4,273)	4,139	(2,234, 6,045)
Bottled Tea	193	18,248	(12,875, 23,621)	18,046	(12,384, 23,707)	226	11,552	(7,058, 16,045)	11,220	(6,939, 15,500)
Plain/Sparkling/Flav. Water	578	108,638	(64,219, 153,057)	117,399	(68,585, 166,214)	643	100,952	(54,311, 147,594)	111,598	(58,049, 165,148)
Diet Energy Drinks	53	22,171	(12,806, 31,536)	22,666	(11,810, 33,522)	57	9,636	(5,668, 13,604)	9,276	(4,990, 13,563)
Diet Sports Drinks	70	63,662	(45,125, 82,199)	61,672	(42,151, 81,192)	81	39,276	(26,923, 51,630)	37,479	(25,394, 49,564)
*Beverage Size*										
Single Serving (≤ 1 L)	1,970	13,468	(11,987, 14,949)	13,890	(12,318, 15,462)	2,312	5,499	(4,837, 6,160)	5,621	(4,910, 6,332)
Multi-pack	832	103,173	(71,663, 134,683)	116,778	(81,453, 152,104)	1,000	77,038	(46,890, 107,187)	83,213	(48,916, 117,511)
Family Size (> 1 L)	792	125,258	(81,519, 168,998)	121,433	(78,090, 164,776)	1,001	64,571	(39,156, 89,986)	60,742	(37,189, 84,294)

KC: King County excluding Seattle. UPC: Universal Product Code. CI: confidence interval. L: liter. Sample is balanced on stores and beverages (defined by the UPC), meaning that unique stores and unique beverages that are present in both the pre and post periods are included in the sample. Mean estimates adjust for clustered standard errors at the UPC level. The combined comparison area includes Oakland County, MI, and Sacramento County, CA.

**Table 2:** Sample of beverages and mean volume sold (liters) by taxable status, type, and size in Seattle and its combined comparison area, 2016-2019.

	Seattle (N stores = 87)					Comparison (N stores = 115)				
	N, UPCs	Pre-tax		Post-tax		N, UPCs	Pre-tax		Post-tax	
		Mean Volume, L	95% CI	Mean Volume, L	95% CI		Mean Volume, L	95% CI	Mean Volume, L	95% CI
**Taxed Beverages**										
Overall	1,439	18,446	(16,134, 20,759)	14,686	(12,845, 16,526)	2,135	7,915	(6,877, 8,954)	7,782	(6,757, 8,808)
*Beverage Type*										
Soda	565	20,976	(16,295, 25,656)	17,538	(13,689, 21,388)	895	9,387	(7,229, 11,544)	9,783	(7,643, 11,923)
Fruit Drinks	376	16,908	(13,336, 20,479)	10,927	(8,740, 13,113)	636	5,412	(4,308, 6,516)	4,508	(3,592, 5,424)
Bottled Coffee	63	8,687	(5,406, 11,968)	8,828	(5,341, 12,315)	75	3,960	(2,483, 5,437)	4,228	(2,507, 5,950)
Bottled Tea	214	11,232	(8,162, 14,302)	9,224	(6,707, 11,740)	260	6,393	(4,639, 8,146)	6,424	(4,477, 8,372)
Energy Drinks	74	15,323	(9,002, 21,644)	15,656	(9,436, 21,877)	74	6,997	(3,886, 10,108)	7,330	(4,066, 10,595)
Sports Drinks	84	38,850	(25,136, 52,563)	28,200	(17,632, 38,768)	114	17,267	(12,190, 22,343)	15,565	(10,432, 20,698)
*Beverage Size*										
Single Serving (≤ 1 L)	871	10,049	(8,266, 11,832)	9,044	(7,518, 10,569)	1,153	3,756	(3,023, 4,490)	3,866	(3,116, 4,616)
Multi-pack	300	20,306	(15,582, 25,029)	18,143	(14,279, 22,006)	529	10,596	(8,095, 13,097)	11,355	(8,824, 13,886)
Family Size (> 1 L)	268	43,657	(34,638, 52,676)	29,152	(22,053, 36,251)	453	15,370	(12,034, 18,706)	13,578	(10,360, 16,796)
**Nontaxed Beverages**										
Overall	3,409	39,007	(31,736, 46,279)	39,987	(32,682, 47,292)	4,859	16,813	(13,111, 20,516)	17,746	(13,837, 21,655)
*Beverage Type*										
Diet Soda	806	41,742	(33,972, 49,511)	46,284	(37,488, 55,080)	1,153	20,313	(15,158, 25,468)	21,936	(16,421, 27,450)
100% Juice/Diet Fruit Drinks	966	13,558	(11,642, 15,473)	10,551	(8,585, 12,517)	1,449	4,831	(4,121, 5,541)	3,752	(3,181, 4,323)
Milk	580	71,060	(40,364, 101,756)	71,196	(41,944, 100,448)	845	27,922	(13,244, 42,600)	29,032	(13,558, 44,507)
Bottled Coffee	71	5,379	(2,498, 8,260)	8,774	(4,316, 13,233)	74	2,472	(1,321, 3,623)	3,901	(2,029, 5,773)
Bottled Tea	190	10,143	(7,447, 12,839)	9,593	(6,779, 12,408)	245	6,183	(4,461, 7,906)	5,956	(4,146, 7,766)
Plain/Sparkling/Flav. Water	526	73,145	(43,198, 103,092)	77,467	(46,205, 108,729)	751	31,118	(15,827, 46,410)	35,266	(19,145, 51,386)
Diet Energy Drinks	50	14,439	(8,289, 20,589)	14,754	(7,877, 21,632)	46	6,670	(4,329, 9,010)	6,692	(3,916, 9,468)
Diet Sports Drinks	59	39,881	(28,028, 51,735)	37,246	(25,916, 48,577)	82	22,178	(16,527, 27,828)	22,133	(15,988, 28,278)
*Beverage Size*										
Single Serving (≤ 1 L)	1,845	10,641	(9,447, 11,835)	10,590	(9,391, 11,788)	2,613	3,161	(2,802, 3,521)	3,303	(2,923, 3,682)
Multi-pack	790	65,055	(45,067, 85,042)	73,333	(52,057, 94,610)	1,122	33,864	(22,603, 45,126)	37,203	(25,430, 48,976)
Family Size (> 1 L)	774	80,039	(55,887, 104,192)	76,025	(52,862, 99,189)	1,124	31,529	(20,283, 42,775)	31,901	(19,912, 43,889)

UPC: Universal Product Code. CI: confidence interval. L: liter. Sample is balanced on stores and beverages (defined by the UPC), meaning that unique stores and unique beverages that are present in both the pre and post periods are included in the sample. Mean estimates adjust for clustered standard errors at the UPC level. The combined comparison area includes Dane County, WI, and Denver County, CO.

### Difference-in-differences in beverage volume sold in King County excluding Seattle

#### Taxed beverages.

**Fig 2:**
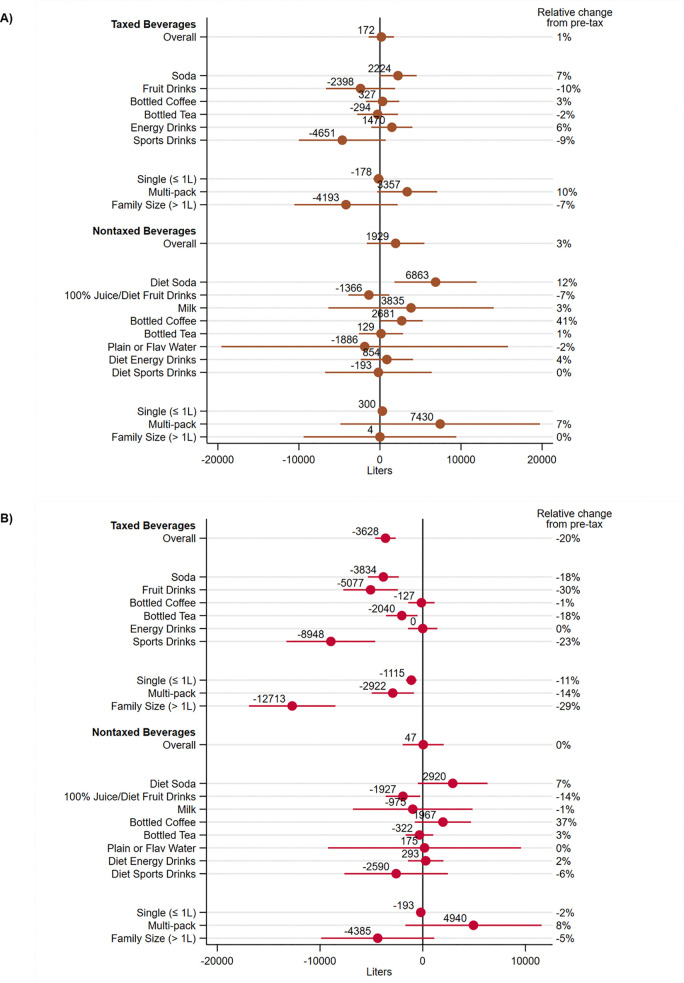
Difference-in-differences in mean volume sold (liters) in (A) King County and (B) Seattle, by taxed status, beverage size, and beverage type, comparing two years before to two years after the Seattle Sweetened Beverage Tax, 2016-2019. Difference-in-differences estimates and 95% confidence intervals reflect the estimated mean change in volume sold for a given beverage in the treated area associated with the timing of the Seattle Sweetened Beverage Tax. Regression models included fixed effects for beverages at the universal product code level and robust standard errors. **(A)** The treated area is King County excluding Seattle and comparison area is the combination of Sacramento, CA, and Oakland, MI, counties. **(B)** The treated area is Seattle and the comparison area is a combination of Dane, WI, and Denver, CO, counties.

The estimate of the Seattle tax effect on mean taxed beverage volume sold in KC relative to the comparison area was 172 liters (95% CI: −1,396, 1,740; P = 0.83) in the two years post-tax compared to two years pre-tax (Table 3). This translates to an approximate 1% change from the mean in the pre-tax period in KC above and beyond changes in the comparison area and does not provide evidence of a tax effect. Estimates of tax effects by beverage type and size similarly reflected little differential change from pre- to post-tax. There was suggestive evidence of small increases for soda (7%; P = 0.06) and multipack beverages (10%; P = 0.08), and a small decrease for sports drinks (−9%, P = 0.09) in KC relative to changes in the comparison area (Fig 2).

**Table 3:** Difference-in-differences in mean volume sold (liters) in Seattle and King County versus comparison areas, by taxed status, beverage size, and beverage type, comparing two years before to two years after the Seattle Sweetened Beverage Tax, 2016-2019.

	King County excluding Seattle (KC) vs. Comparison Area					Seattle vs. Comparison Area					Seattle DD v. KC DD		
	Pre-tax Mean	DD Estimate	95% CI	P value	Percent change from pre-tax	Pre-tax Mean	DD Estimate	95% CI	P value	Percent change from pre-tax	DDD Estimate	95% CI	P value
**Taxed Beverages**													
Overall	27,324	172	(−1,396, 1,740)	0.83	1%	18,446	**−3,628**	**(−4,622, −2,634)**	**< 0.001**	**−20%**	**−3,800**	**(−5,656, −1,944)**	**< 0.001**
*Beverage Type*													
Soda	30,604	2,224	(−87, 4,535)	0.06	7%	20,976	**−3,834**	**(−5,333, −2,336)**	**< 0.001**	**−18%**	**−6,058**	**(−8,811, −3,305)**	**< 0.001**
Fruit Drinks	24,290	−2,398	(−6,659, 1,862)	0.27	−10%	16,908	**−5,077**	(−7,731, −2,423)	**< 0.001**	**−30%**	−2,679	(−7,694, 2,336)	0.30
Bottled Coffee	12,356	327	(−1,730, 2,384)	0.75	3%	8,687	−127	(−1,403, 1,149)	0.84	−1%	−454	(−2,860, 1,952)	0.71
Bottled Tea	15,917	−294	(−2,818, 2,230)	0.82	−2%	11,232	**−2,040**	(−3,579, −500)	**0.01**	**−18%**	−1,746	(−4,697, 1,205)	0.25
Energy Drinks	24,970	1,470	(−1,065, 4,006)	0.25	6%	15,323	0	(−1,427, 1,426)	0.99	0%	−1,471	(−4,364, 1,423)	0.32
Sports Drinks	54,260	−4,651	(−10,020, 718)	0.09	−9%	38,850	**−8,948**	**(−13,276, −4,620)**	**< 0.001**	**−23%**	−4,297	(−11,163, 2,569)	0.22
*Beverage Size*													
Single Serving (≤ 1 L)	14,044	−178	(−820, 464)	0.59	−1%	10,049	**−1,115**	**(−1,643, −588)**	**< 0.001**	**−11%**	**−937**	**(−1,768, −106)**	**0.03**
Multi-pack	34,175	3,357	(−347, 7,060)	0.08	10%	20,306	**−2,922**	**(−4,979, −864)**	**0.01**	**−14%**	**−6,279**	**(−10,511, −2,047)**	**0.004**
Family Size (> 1 L)	61,527	−4,193	(−10,569, 2,183)	0.20	−7%	43,657	**−12,713**	**(−16,923, −8,502)**	**< 0.001**	**−29%**	**−8,520**	**(−16,152, −888)**	**0.03**
**Nontaxed Beverages**													
Overall	58,869	1,929	(−1,628, 5,487)	0.29	3%	39,007	46	(−1,950, 2,043)	0.96	0%	−1,883	(−5,962, 2,196)	0.37
*Beverage Type*													
Diet Soda	58,601	**6,863**	**(1,793, 11,932)**	**0.01**	**12%**	41,742	2,920	(−471, 6,310)	0.09	7%	−3,943	(−10,039, 2,153)	0.21
100% Juice/Diet Fruit Drinks	18,928	−1,366	(−3,899, 1,168)	0.29	−7%	13,558	**−1,927**	**(−3,605, −249)**	**0.02**	**−14%**	−561	(−3,599, 2,476)	0.72
Milk	114,180	3,835	(−6,368, 14,038)	0.46	3%	71,060	−975	(−6,800, 4,850)	0.74	−1%	−4,810	(−16,552, 6,932)	0.42
Bottled Coffee	6,562	**2,681**	**(81, 5,281)**	**0.04**	**41%**	5,379	1,967	(−773, 4,706)	0.16	37%	−714	(−4,469, 3,040)	0.71
Bottled Tea	18,248	129	(−2,594, 2,853)	0.93	1%	10,143	−322	(−1,664, 1,020)	0.64	−3%	−451	(−3,482, 2,579)	0.77
Plain/Sparkling/Flav. Water	108,638	−1,886	(−19,567, 15,796)	0.83	−2%	73,145	175	(−9,224, 9,573)	0.97	0%	2,060	(−17,950, 22,070)	0.84
Diet Energy Drinks	22,171	854	(−2,370, 4,079)	0.60	4%	14,439	293	(−1,437, 2,024)	0.74	2%	−561	(−4,192, 3,070)	0.76
Diet Sports Drinks	63,662	−193	(−6,774, 6,389)	0.95	0%	39,881	−2,590	(−7,631, 2,451)	0.31	−6%	−2,397	(−10,639, 5,845)	0.57
*Beverage Size*													
Single Serving (≤ 1 L)	13,468	300	(−250, 849)	0.29	2%	10,641	−193	(−598, 213)	0.35	−2%	−492	(−1,175, 190)	0.16
Multi-pack	103,173	7,430	(−4,880, 19,740)	0.24	7%	65,055	4,940	(−1,687, 11,567)	0.14	8%	−2,490	(−16,465, 11,484)	0.73
Family Size (> 1 L)	125,258	4	(−9,417, 9,425)	0.99	0%	80,039	−4,385	(−9,896, 1,125)	0.12	−5%	−4,389	(−15,298, 6,520)	0.43

DD: difference-in-differences. DDD: triple difference. CI: confidence interval. UPC: Universal Product Code. L: liter.

Balanced sample of stores and beverages (defined by the UPC). Analyses use a linear DD regression model adjusted for UPC fixed effects and with standard errors clustered at the UPC level. Beverages with unknown taxed status or unknown beverage category are omitted. The comparison area for KC is the combined area of Sacramento County, CA, and Oakland County, MI, and the comparison area for Seattle is the combined area of Dane County, WI, and Denver County, CO.

When restricting the sample to balanced UPCs sold in the last year preceding the tax and the first year after the tax, some tax effect estimates showed greater increases compared to the primary results over the four-year period (S1 Table). While there was little tax effect on the mean volume sold of taxed beverages overall in the first year in KC (603 liters [95% CI: −134, 1,339; P = 0.11], a 7% increase), there was some evidence of increased volume sold for soda (15%; P = 0.03), energy drinks (20%; P = 0.02), and multipack beverages (26%; P = 0.004) in association with the tax.

In the unbalanced sample of UPCs, we retained UPCs that were present only in one of the pre- or post-tax periods and we adjusted for beverage type and size. Several tax effect estimates differed from those in the primary balanced sample (S2 Table). The tax effect for an average UPC among taxed beverages overall was more positive, at 2,212 liters (95% CI: 675, 3,748; P = 0.01), reflecting an approximate 16% increase in taxed beverages sold in KC relative to the comparison area. We also observed increases for soda (44%; P = 0.003) and multipack beverages (17%; P = 0.01) in association with the tax.

#### Nontaxed beverages.

The estimate of the Seattle tax on mean volume sold of nontaxed beverages in KC was 1,929 liters (95% CI: −1,628, 5,487; P = 0.29), which was only 3% higher than the pre-tax period after accounting for changes in the comparison area (Table 3). Tax effect estimates were generally small across nontaxed beverage types and sizes except for increases in volume sold of diet soda (12%; P = 0.01) and diet/unsweetened bottled coffee (41%; P = 0.04) relative to changes in the comparison area (Fig 2).

In the analysis restricted to one year before and after tax implementation, tax effect estimates were generally more positive than in the primary analysis (S1 Table). The effect of the tax on volume sold in KC was 2,069 liters (95% CI: 237, 3,900; P = 0.03) for nontaxed beverages overall, a 13% increase relative to the comparison area. We also observed increases for diet soda (24%; P = 0.003), milk (26%; P = 0.03), diet/unsweetened bottled coffee (70%; P = 0.01), and nontaxed single serving sizes (23%; P < 0.001) in association with the tax.

When we estimated the tax effect estimate in the unbalanced sample of UPCs, the tax effect in KC was more positive than the balanced sample estimate (S2 Table). We observed an increase of 3,064 liters (95% CI: 130, 5,999; P = 0.04) for nontaxed beverages overall in KC above and beyond the change in the comparison area, which approximated a 31% increase from the pre-tax period. We observed increases for milk (35%; P = 0.02), diet/unsweetened bottled coffee (61%; P = 0.07), and nontaxed family size beverages (10%; P = 0.08) as well.

### Difference-in-differences in beverage volume sold in Seattle

#### Taxed beverages.

Among taxed beverages overall, the effect of the tax on mean volume sold in Seattle was −3,628 liters (95% CI: −4,622, −2,634; P < 0.001) within the two years post-tax (Table 3). This amounts to a 20% decrease in taxed volume sold in Seattle above and beyond the change in the comparison area. Tax effect estimates were similarly negative and large across beverage types and sizes, with notable decreases for soda (−18%; P < 0.001), fruit drinks (−30%; P < 0.001), sports drinks (−23%; P < 0.001), and family size beverages (−29%; P < 0.001) in Seattle relative to the comparison area. No change was observed for bottled coffee (−1%; P = 0.84) and energy drinks (0%; P = 0.99) (Fig 2).

In analyses restricted to one year before and after tax implementation, tax effect estimates for overall taxed beverages and by type and size were similar to those in the primary analysis (S1 Table). In the unbalanced sample of UPCs, estimates of the tax effect were also generally negative and similar to those in primary analysis (S2 Table).

#### Nontaxed beverages.

The effect of the tax on the mean volume sold of nontaxed beverages in Seattle was 46 liters (95% CI: −1,950, 2,043; P = 0.96), approximating 0% change in volume sold in the two years post-tax (Table 3). Across beverage types and sizes, tax effect estimates were generally small and suggested minimal change associated with the tax. An exception was a decrease in volume sold for 100% fruit juices/diet fruit drinks (−14%; P = 0.02) relative to the comparison area (Fig 2).

When we restricted the sample to purchases in the one year preceding and one year following tax implementation, tax effect estimates suggested an increase in nontaxed volume sold in Seattle (S1 Table). The effect of the tax for nontaxed beverages overall was 1,310 liters (95% CI: 386, 2,235; P = 0.01), a 14% increase from the pre-tax period in Seattle. Estimates suggested increases for diet soda (15%; P = 0.03), milk (19%; P = 0.06), and diet/unsweetened bottled coffee (60%; P = 0.06), as well as increases for nontaxed single serving sizes (18%; P < 0.001) and nontaxed multipack sizes (15%; P = 0.08). In the unbalanced sample of UPCs, estimates of the tax effect were similar to those in the primary analysis sample, except for a large increase for diet/unsweetened bottled coffee (82%; P = 0.06) (S2 Table).

### Triple difference in beverage volume sold in Seattle relative to King County excluding Seattle

For taxed beverages overall, the triple difference estimate suggests that the effect of the tax on mean volume sold in Seattle was more negative than the effect of the tax in KC by −3,800 liters (95% CI: −5,656, −1,944; P < 0.001), on average, in the two years post-tax (Table 3). Since we observed evidence of a tax-related decrease in volume sold in Seattle from the DD analyses, but not in KC, the triple difference estimate is similar in magnitude to the Seattle DD estimate. Among specific beverage types and sizes, there were larger tax-related decreases in Seattle relative to KC for taxed soda (DDD: −6,058 liters; 95% CI: −8,811, −3,305; P < 0.001), taxed single serving sizes (DDD: −937 liters; 95% CI: −1,768, −106; P = 0.03), taxed multipack sizes (DDD: −6,279 liters; 95% CI: −10,511, −2,047; P = 0.004), and taxed family sizes (DDD: −8,520 liters; 95% CI: −16,152, −888; P = 0.03). Among nontaxed beverages, triple difference estimates were generally negative and small in magnitude; altogether, there was no evidence that the tax effect for nontaxed beverages in Seattle differed from the tax effect in KC.

## Discussion

In this quasi-experimental study of the Seattle Sweetened Beverage Tax, we did not find evidence that the tax was associated with changes in SSB purchasing in communities nearby but not bordering Seattle (i.e., KC) two years post-tax. Primarily, volume sold of taxed beverages in KC over time did not differ meaningfully from volume sold in the matched comparison area over the same period. This suggests that any health risk signaling effects of the tax that might influence behavior did not spillover into nearby communities and meaningfully influence beverage purchasing. In Seattle, we observed an approximate decrease of 20% in taxed beverage volume sold in association with the tax relative to the comparison area. We observed this tax effect even though the defined Seattle area in this study included immediate bordering cities. Our findings in Seattle align with the 22% reduction reported in a previous study that used a similar dataset but different comparison area and model specifications [14].

Our overall finding that Seattle’s SSB was not associated with changes in taxed beverage purchasing in KC has mixed support from results of other Seattle SSB tax studies. First, two recent studies observed tax impacts on body mass index change in Seattle relative to nearby counties [8,41]. Our study was consistent with these findings in that they suggest spillover was not present to the extent that it masked observable tax impacts in Seattle when compared to nearby counties. On the other hand, our study is less consistent with a study by Saelens and colleagues of SSB consumption among families with lower income in Seattle and near-bordering comparison areas that found no evidence of a tax impact and instead found SSB consumption to decrease in both Seattle and comparison areas [16,17]. Since there is strong evidence that the tax in Seattle resulted in higher prices of taxed beverages and decreased purchasing of these beverages in Seattle [14,15], the finding from Saelens and colleagues that SSB consumption decreased similarly in nearby areas is consistent with the idea of spillover on purchasing and consumption in KC, but is inconsistent with our null findings for spillover effects. In other work to explore why Saelens and colleagues found decreases in both Seattle and comparison areas, a qualitative study found that some comparison area participants reported decreasing their SSB consumption in part due to exposure to the price and messaging of the tax [42]. If spillover effects were experienced among lower but not higher income households, such as those in the study by Saelens and colleagues, this may explain our different findings. This is possible since small, independent stores (e.g., corner stores) are underrepresented in scanner data used in our study [43], and they tend to be the primary source for SSB purchases among residents of lower-income neighborhoods [43–45]. It is also possible that tax spillover effects were indeed negligible, and consistent by income, and that the SSB consumption results from the above studies were instead influenced by self-reporting biases.

Beyond the Seattle SSB tax context, there is limited research on spillover effects in nearby areas. Our findings are consistent with a study from Cook County, IL, that found no evidence of health risk signaling of a SSB tax [26] and thus no mechanism for spillover effects in nearby areas. In the study, researchers assessed how purchases of taxed beverages changed after a SSB tax was briefly implemented and then repealed, providing a unique opportunity to understand the extent to which price and health risk signaling effects influenced behavior [26]. Since beverage purchasing returned to pre-tax levels soon after the repeal, it is unlikely that the tax had a health risk signaling effect, i.e., consumers did not continue to purchase less due to the tax conveying health risk information about SSB, and therefore, it is unlikely that signaling effects spilled over into nearby areas around Cook County. On the other hand, our findings are inconsistent with a study that supports the hypothesis of spillover effects in that nearby versus distal comparison areas tend to estimate smaller tax impacts [12], but this has not been systematically evaluated across the SSB tax impact literature. One study that contrasts with this hypothesis that areas near a tax may trend more similarly than distal areas, and thus offers some support for our null finding of spillover effects, is a study of Berkeley’s tax, which observed a larger tax effect on SSB purchasing when compared to in-state controls versus out-of-state controls [31]. However, the study omitted nearby areas around the tax from the analysis, and therefore the findings say more about state-level secular trends than spillover within a shared media market.

While our primary finding did not provide evidence of tax spillover effects in the form of reduced purchasing in KC, it was unexpected to find suggestive evidence of small increases in purchasing of some taxed beverages in KC. In the two years post-tax, there were non-statistically significant increases in volume sold for taxed soda (7%) and multipack (10%) beverages in KC that became larger and statistically significant in analyses that examined changes one-year post-tax and in an unbalanced sample. These increases could be consistent with cross-border shopping, with the hypothesis that purchasing increased because shoppers avoided the tax. While moderate cross-border shopping has been observed in bordering areas of several local SSB taxes, such as Oakland and Philadelphia, it was not observed in bordering areas of Seattle [14,36,46–48]. In our study, however, we examined purchases in non-bordering areas around Seattle and observed increases in volume sold for soda and multipack beverages in KC relative to the comparison area, particularly in the first year post-tax. Shoppers may have been more motivated early on to travel to non-bordering cities to avoid the tax. Yet, there are aspects of these findings that are less consistent with a cross-border shopping hypothesis. First, bordering areas most susceptible to cross-border shopping were not part of the KC treated area in our analysis, and instead were included in the Seattle treated area. Second, we observed moderate increases in purchasing of some nontaxed beverages in KC as well, such as diet soda in the first year (24%) and combined two years (12%), and diet single serving size beverages in the first year post tax (24%). We observed similar patterns in Seattle for these nontaxed beverages. To better understand these findings, we performed post-hoc event study analyses to explore how purchasing changed from one to two years post-tax in KC and Seattle. Relative to the KC comparison areas, results suggested that volume sold of taxed and nontaxed beverages increased slightly in KC in the first year and decreased slightly in the second year, but these were not statistically significant (S2 Fig). It is unclear why both taxed and nontaxed beverages would increase in KC, and we were unable to assess individual-level changes in shoppers’ purchasing that could bring light to this. In the Seattle taxed area, one potential explanation for the increases we observed for nontaxed beverages like diet soda is that some shoppers may have switched from purchasing taxed to nontaxed beverages (i.e., substitution), as was suggested in a previous Seattle study [14]. However, this reasoning would not easily extend to the increase in nontaxed beverages that we observed in KC.

Unlike previous studies of tax impacts on purchasing in Seattle, we additionally estimated tax effects using an unbalanced sample of beverages. Our results differed from our primary analysis with a balanced sample in that we observed increased purchasing for overall taxed beverages (16%), taxed soda (44%), taxed multipack beverages (17%) as well as overall nontaxed beverages (31%) in KC relative to the comparison area. Results appear to be largely driven by products new to the market in the post-tax period, suggesting shoppers purchased more new beverage products within the same stores over time. Cross-border shopping is an unlikely explanation for these specific results because we would instead expect to observe increases in volume sold of taxed beverages previously on the market (i.e., in the balanced sample). It is possible that in-store promotions and marketing changed in response to the tax and contributed to this finding. For instance, in-store SSB advertising tends to decrease in the taxed area as producers attempt to reduce costs [49–52]. Consequently, in-store advertising may have increased in stores beyond Seattle to offset losses. In a study of supermarket interior marketing displays before and after Seattle’s tax, non-statistically significant increases for taxed and nontaxed SSB were observed in the South King County comparison area relative to Seattle [52]. The extent to which such advertising applies to new products is unclear and warrants further study.

### Limitations

First, treatment assignment of stores in Seattle and KC involved misclassification because we were limited to the store’s county and three digits ZIP code as geographic identifiers which do not perfectly align with Seattle city boundaries. As noted earlier, this type of misclassification would bias estimates of a tax effect toward the null, producing underestimates of relative increases or decreases in volume sold in the taxed areas and neighboring areas. In terms of our results, this suggests that average taxed volume sold in stores within the Seattle city border may have decreased to a greater degree than we observed, and that taxed and nontaxed volume sold in KC stores closer to Seattle may have increased to a greater degree than we observed (both of which were non-statistically significant increases). Because we observed a similar magnitude of change in volume sold in Seattle to a previous study with more precise treatment assignment [14], this also suggests that cross-border shopping in the areas immediately bordering the tax had little influence on our results.

Second, the store sample is not representative of all retailers in the treated and comparison areas; therefore, we examine within-UPC changes in volume sold within the same stores over time to minimize bias related to store participation and product availability. Convenience stores and other small/independent stores are underrepresented in the scanner data, and dollar stores are absent from the data. Further, we are unable to analyze volume sold in food service establishments or vending machines. The results of this study are more generalizable to SSB purchasing patterns in larger retailers and the types of consumers who predominantly shop there. In addition, our findings may not be generalizable to rural areas.

Third, we cannot control for potential bias due to time-varying, market-specific factors because we selected comparison areas in different media markets from the treated areas. While a synthetic control study design with many weighted comparison areas would have diminished this concern, the burden of classifying thousands of unique beverages in each comparison area was too high to justify this approach. Instead, we examined weekly trends in the two years pre-tax to assess plausibility of parallel trends.

Fourth, some tax effect estimates for beverage categories had wide confidence intervals, suggesting limited statistical power in some cases. For example, we were unable to more precisely assess whether estimated changes in volume sold of taxed soda and taxed multipack beverages in KC were attributed to the tax. Given this limitation in combination with multiple tests performed across beverage type and size categories, tax effect estimates for beverage type and sizes are interpreted cautiously in the context of the magnitude of the change and confidence interval, and thus are more exploratory than the primary analysis of all taxed and nontaxed beverages. Finally, with the small number of treated and comparison areas in the study, as is the case for most SSB tax studies, we were unable to adjust for clustering at the level of treatment and thus standard errors may be underestimated [53].

## Conclusion

This quasi-experimental study is among the first to directly assess the extent to which SSB tax spillover effects may occur for beverage purchasing in communities beyond cross-border shopping. We did not find evidence of spillover effects in the form of reduced volume sold in communities near but not bordering the taxed city. Suggestive evidence of increased volume sold for regular and diet soda, as well as regular multipack beverages, raise questions about intensified beverage marketing in nearby areas post-tax. In the absence of additional evidence, this study supports efforts towards adopting state or national SSB taxes to increase the public health benefit of this policy. Opportunities for future research include investigation of spillover of tax signaling effects in other local SSB tax settings, including effects on purchasing and self-reported consumption. Future research is also needed to understand changes to media and beverage marketing exposure in nontaxed areas around a SSB tax.

## Supporting information

S1 File Comparison area selection and statistical equations. This document provides additional detail about the methods for identifying and selecting the comparison areas and the equations and interpretations of the coefficients in the statistical models.
(DOCX)

S1 Table Difference-in-differences in mean volume sold (liters) in Seattle and King County versus comparison areas, by taxed status, comparing one year before to one year after the Seattle Sweetened Beverage Tax, 2017–2018. This table presents results from a secondary analysis that examined changes in beverage volume sold in the first year post-tax compared to one year preceding the tax.
(DOCX)

S2 Table Difference-in-differences in mean volume sold (liters) in Seattle and King County versus comparison areas, by taxed status, comparing two years before to two years after the Seattle Sweetened Beverage Tax in an unbalanced sample of UPCs, 2016–2019. This table presents results from a secondary analysis that did not restrict the sample to a panel dataset.
(DOCX)

S1 Fig Event study plots of the monthly mean volume sold (liters) of taxed and nontaxed beverages in King County excluding Seattle (KC) and Seattle relative to comparison areas in the two years preceding the Seattle Sweetened Beverage Tax, 2016–2017. This figure displays the coefficients of *a priori* event studies comparing the differences in the monthly beverage volume sold in the treated area versus comparison area prior to tax implementation. This analysis helped assess the extent to which the parallel trends assumption was reasonable in the primary differences-in-differences analysis.
(DOCX)

S2 Fig Difference-in-differences in annual mean volume sold (liters) of taxed and nontaxed beverages in King County excluding Seattle (KC) and Seattle relative to the comparison areas from two years before and after the Seattle Sweetened Beverage Tax, 2016–2019. The figure displays the coefficient results of an event study comparing the differences in annual beverage volume in the treated vs. comparison areas relative to the last year preceding tax implementation.
(DOCX)
