# Supplementary material for: Investigation of spillover effects of a sugar-sweetened beverage tax on beverage purchasing in a nearby, non-taxed area: A quasi-experimental, difference-in-differences analysis
Source: PLoS One. 2026 Feb 4;21(2):e0340577. doi: 10.1371/journal.pone.0340577 (PMC12872015; doi:10.1371/journal.pone.0340577)
Supplement: S1 File — This document provides additional detail about the methods for identifying and selecting the comparison areas and the equations and interpretations of the coefficients in the statistical models. (DOCX) [file pone.0340577.s001.docx]

**Comparison area selection**

To select the comparison areas, we first calculated the Mahalanobis distance^1^ between each treated area and nonrural US counties to identify well-matched candidates. We included the following variables in the Mahalanobis distance: American Community Survey (ACS) 2017 five-year estimates of county-level population size and density, age distribution, race and ethnicity distribution (non-Hispanic Asian, non-Hispanic Black, non-Hispanic white, and Hispanic), educational attainment, median household income, proportion living in poverty, and proportion of residents who rent^2^. For the treated areas, we calculated the above variables using combined zip code tabulation areas to align with the treatment assignment in the retail scanner data (980- zip codes for KC and 981- zip codes for Seattle).

Second, from the top 30 closest matched counties, we excluded those with a history of passing or proposing a sweetened beverage tax either in the county or in the county’s Census-defined Core-Based Statistical Area during the study period. To align political contexts, we excluded counties in which Democratic votes were the minority for the 2016 Presidential Election.

Third, we estimated a proxy for pre-tax sweetened beverage consumption levels by calculating per capita volume sold of carbonated beverages in each area, using the “carbonated beverages” product module in the NielsenIQ retail scanner data. It was important to assess pre-tax per capita volume sold because large changes in an area could reflect increases in retailer participation in the dataset rather than increases in actual volume sold.

We considered all the above measures to select three top comparison area candidates for each treated area. Among the top three comparison area candidates for each treated area, we assessed evidence of pre-trends in the volume sold of taxed beverages by plotting volume sold over time in the pre-tax period. We found that two comparison areas trended similarly with each of the treated areas. Therefore, we decided to create a combined comparison area of the two counties for each exposed area.

The comparison area for KC was the combination of Sacramento County, CA, and Oakland County, MI, Based on 2017 ACS estimates, both counties and KC were metropolitan areas with populations ranging 1.2-1.5 million and similar age distributions (e.g., 18-24-year-olds accounted for 22% in KC, 25% in Sacramento County, and 22% in Oakland County) (S1 File Table). Relative to KC, Sacramento County was more racially and ethnically diverse and Oakland County was less, though the proportion of non-Hispanic Black residents is Oakland County was notably larger (14% v. 5%). Oakland County was more similar to KC than Sacramento County with respect to educational attainment, median household income, and poverty.

The comparison area for Seattle was the combination of Dane County, WI, and Denver County, CO. The Seattle treated area (defined by 981- zip codes) had a larger population than Dane and Denver counties (931,961 v. 522,837 and 678,467, respectively) and similar age distributions in 2017 (S1 File Table). Compared to Seattle, Dane County had a larger non-Hispanic white population (80% v. 61%), Denver County had a larger Hispanic population (31% v. 9%), and both counties had smaller non-Hispanic Asian populations (6% and 4% v. 15%). Under the parallel trends assumption, we assumed that observed and unobserved differences between demographic measures in treated and comparison areas would trend similarly over the study period and thus would not confound the association between tax implementation and SSB purchasing.

**S1 File Table.** Demographic Measures used in the Mahalanobis Distance Calculation for Comparison Areas and Treated Areas, American Community Survey 2017 Five-Year Estimates.

|  | **King County excluding Seattle (KC), WA**** | **Sacramento County, CA** | **Oakland County, MI** | **Seattle, WA**** | **Dane County, WI** | **Denver County, CO** |
| --- | --- | --- | --- | --- | --- | --- |
| Core-based Statistical Area* | NA | Sacramento-Roseville-Arden-Arcade, CA | Detroit-Warren-Dearborn, MI | NA | Madison, WI | Denver-Aurora-Lakewood, CO |
| Total Population | 1,264,571 | 1,495,400 | 1,241,860 | 931,961 | 522,837 | 678,467 |
| Population Density (persons per sq. mi.) | 789.1 | 1,549.8 | 1,431.5 | 6,492.7 | 437.0 | 4,425.6 |
| Under 18 years (%) | 23.7 | 24.2 | 21.8 | 16.92 | 21.9 | 20.4 |
| 18-24 years (%) | 22.2 | 24.6 | 20.8 | 30.72 | 29.4 | 30.7 |
| Over 65 years (%) | 12.1 | 13.0 | 15.5 | 12.67 | 12.3 | 11.2 |
| Non-Hispanic Asian (%) | 18.0 | 15.1 | 6.8 | 14.7 | 5.6 | 3.5 |
| Non-Hispanic Black (%) | 4.6 | 9.5 | 13.6 | 7.9 | 5.0 | 9.2 |
| Non-Hispanic White (%) | 61.1 | 45.8 | 72.9 | 61.2 | 80.2 | 53.6 |
| Hispanic (%) | 10.0 | 22.8 | 3.9 | 8.9 | 6.3 | 30.5 |
| Less than a high school degree (%) | 7 | 13.0 | 6.3 | 7.9 | 4.4 | 13.3 |
| Bachelor's degree or higher (%) | 46.3 | 29.9 | 45.7 | 54.2 | 50.0 | 46.5 |
| Median household income ($) | 88533 | 60239 | 73369 | 75556 | 67631 | 60098 |
| Below the FPL (%) | 5.8 | 12.6 | 6.3 | 7.4 | 5.87 | 11.1 |
| Residents who rent (%) | 21.9 | 32.0 | 17.8 | 31.0 | 23.2 | 32.1 |

FPL= Federal Poverty Level.

*For information only; not used in Mahalanobis distance matching.

**The treated areas of KC and Seattle are defined by three-digit zip code tabulation areas (980- and 981-, respectively) to align with the treatment assignment in the retail scanner data. Therefore, population counts and demographic measures differ from the estimates defined by city and county borders.

**Difference-in-differences equations**

**Differences in Differences**

We used two linear DD regression models to estimate the mean difference in liters sold associated with the SSB tax from 2 years before and after implementation. The comparison areas are specific for each treated area: Sacramento and Oakland counties for King County excluding Seattle (KC), and Dane and Denver counties for Seattle. The models will compare the changes in Seattle v. comparison area, and KC v. comparison area:

1. *Y_KC_it_ = β_0_ + β_1_[Time]_t_ + β_2_[KC]_i_ + β_3_[Time*KC]_it_ + ε_it_,*
2. *Y_Seattle_it_ = β_0_ + β_1_[Time_i_]_t_ + β_2_[Seattle]_i_ + β_3_[Time*Seattle]_it_ + ε_it_*

Where Y*_it_* is the mean outcome *i* at time *t,* and *Time* is set to a value of 1 for post-tax observations and 0 for pre-tax observations. *KC* is an indicator variable set to 1 for purchases in KC and 0 for purchases in the comparison are. Likewise, *Seattle* is set to 1 for purchases in Seattle and 0 for the comparison area. *β_3_* is the coefficient of interest in each model, representing the change in the mean volume purchased in the treated area above and beyond the change in the comparison area over the same time. Fixed effects at the UPC-level were used to adjust for time-invariant characteristics specific to each beverage.

**Triple Difference**

The triple difference (DDD) estimates the degree to which Seattle experiences a larger effect of the tax than in KC. The benefit of estimating this model is to be able to test whether the DDD coefficient is different from 0, meaning the effect of the tax in Seattle was different than the effect of the tax in KC.

We fit the following linear regression model:

*Y_it_ = β_0_ + β_1_[Time]_t_ + β_2_ [Group] + β_3_ [Treat] + β_4_[Time*Group] + β_5_[Time*Treat] + β_6_[Group*Treat] + β_7_[Time*Group*Treat] ε,*

Where Y*_it_* is the mean outcome *i* at time *t,* and *Time* is set to a value of 1 for post-tax observations and 0 for pre-tax observations. *Treat* is a dummy variable set to 1 for Seattle and KC areas, and 0 for comparison areas. Likewise, *Seattle* is set to 1 for Seattle and its comparison area of Dane and Denver counties, 0 for KC and its comparison area of Sacramento and Oakland counties. Fixed effects were applied as described above.

**References**

1. Kantor D. MAHAPICK: Stata module to select matching observations based on a Mahalanobis distance measure. Published online February 2006. Accessed June 27, 2023. https://ideas.repec.org/c/boc/bocode/s456703.html

2. U.S. Census Bureau. 2015-2019 American Community Survey 5-Year Estimates. Accessed March 29, 2022. https://www.census.gov/acs/www/data/data-tables-and-tools/narrative-profiles/2019/report.php?geotype=place&state=53&place=63000
