# Supplementary material for: Investigation of spillover effects of a sugar-sweetened beverage tax on beverage purchasing in a nearby, non-taxed area: A quasi-experimental, difference-in-differences analysis
Source: PLoS One. 2026 Feb 4;21(2):e0340577. doi: 10.1371/journal.pone.0340577 (PMC12872015; doi:10.1371/journal.pone.0340577)
Supplement: S1 Table — This table presents results from a secondary analysis that examined changes in beverage volume sold in the first year post-tax compared to one year preceding the tax. (DOCX) [file pone.0340577.s002.docx]

**S1 Table**. Difference-in-differences in mean volume sold (liters) in Seattle and King County versus comparison areas by taxed status comparing one year before to one year after the Seattle Sweetened Beverage Tax 2017-2018.

|  | King County excluding Seattle (KC) vs. Comparison Area | | | | | Seattle vs. Comparison Area | | | | |  |
| --- | --- | --- | --- | --- | --- | --- | --- | --- | --- | --- | --- |
|  | Pre-tax mean | DD Estimate | 95% CI | *P* value | Percent change from pre-tax | Pre-tax mean | DD estimate | 95% CI | *P* value | Percent change from pre-tax |  |
| **Taxed Beverages** |  |  |  |  |  |  |  |  |  |  |  |
| Overall | 8,172 | 603 | (-134, 1,339) | 0.11 | 7% | 4,552 | **-994** | **(-1,366, -622)** | **< 0.001** | **-22%** |  |
| *Beverage Type* |  |  |  |  |  |  |  |  |  |  |  |
| Soda | 9,137 | **1,330** | (141, 2,519) | **0.03** | **15%** | 5,047 | **-1,089** | **(-1,558, -620)** | **< 0.001** | **-22%** |  |
| Fruit Drinks | 7,054 | -306 | (-2,167, 1,555) | 0.75 | -4% | 3,801 | -1,222 | (-2,312, -131) | 0.03 | -32% |  |
| Bottled Coffee | 2,897 | 534 | (-224, 1,291) | 0.17 | 18% | 2,117 | -70 | (-524, 384) | 0.76 | -3% |  |
| Bottled Tea | 5,289 | 86 | (-1,019, 1,192) | 0.88 | 2% | 3,331 | **-801** | **(-1,410, -193)** | **0.01** | **-24%** |  |
| Energy Drinks | 7,552 | **1,516** | (238, 2,794) | **0.02** | **20%** | 4,585 | 224 | (-424, 871) | 0.50 | 5% |  |
| Sports Drinks | 18,192 | -772 | (-3,188, 1,644) | 0.53 | -4% | 10,572 | **-3,061** | **(-4,298, -1,823)** | **< 0.001** | **-29%** |  |
| *Beverage Size* |  |  |  |  |  |  |  |  |  |  |  |
| Single Serving (≤ 1 liter) | 3,940 | **355** | (13, 697) | **0.04** | **9%** | 2,358 | **-223** | **(-411, -35)** | **0.02** | **-9%** |  |
| Multi-pack | 9,612 | **2,468** | (811, 4,125) | **0.004** | **26%** | 5,190 | **-779** | **(-1,391, -167)** | **0.01** | **-15%** |  |
| Family Size (> 1 liter) | 18,394 | -543 | (-3,561, 2,475) | 0.72 | -3% | 10,351 | **-3,518** | **(-5,205, -1,831)** | **< 0.001** | **-34%** |  |
| **Nontaxed Beverages** |  |  |  |  |  |  |  |  |  |  |  |
| Overall | 16,256 | **2,069** | (237, 3,900) | **0.03** | **13%** | 9,481 | **1,310** | **(386, 2,235)** | **0.01** | **14%** |  |
| *Beverage Type* |  |  |  |  |  |  |  |  |  |  |  |
| Diet Soda | 16,315 | **3,844** | (1,273, 6,415) | **0.003** | **24%** | 11,013 | **1,671** | **(124, 3,219)** | **0.03** | **15%** |  |
| 100% Juice/Diet Fruit Drinks | 5,180 | 479 | (-649, 1,608) | 0.41 | 9% | 3,186 | 204 | (-555, 963) | 0.60 | 6% |  |
| Milk | 24,951 | **6,382** | (529, 12,235) | **0.03** | **26%** | 15,754 | 2,992 | (-179, 6,164) | 0.06 | 19% |  |
| Bottled Coffee | 1,795 | **1,266** | (328, 2,203) | **0.01** | **70%** | 1,487 | 899 | (-36, 1,833) | 0.06 | 60% |  |
| Bottled Tea | 5,456 | 403 | (-711, 1,516) | 0.48 | 7% | 3,055 | 91 | (-485, 667) | 0.76 | 3% |  |
| Plain/Sparkling/Flav. Water | 36,415 | -1,736 | (-10,514, 7,043) | 0.70 | -5% | 17,713 | 1,996 | (-2,021, 6,013) | 0.33 | 11% |  |
| Diet Energy Drinks | 5,959 | 1,345 | (-261, 2,951) | 0.10 | 23% | 4,088 | 665 | (-283, 1,612) | 0.17 | 16% |  |
| Diet Sports Drinks | 20,482 | 2,560 | (-283, 5,403) | 0.08 | 12% | 12,109 | -575 | (-2,485, 1,335) | 0.55 | -5% |  |
| *Beverage Size* |  |  |  |  |  |  |  |  |  |  |  |
| Single Serving (≤ 1 liter) | 3,358 | **756** | (512, 1,000) | **< 0.001** | **23%** | 2,286 | **414** | **(238, 590)** | **< 0.001** | **18%** |  |
| Multi-pack | 31,969 | 3,305 | (-2,813, 9,424) | 0.29 | 10% | 17,739 | 2,577 | (-281, 5,434) | 0.08 | 15% |  |
| Family Size (> 1 liter) | 32,019 | 4,338 | (-748, 9,424) | 0.10 | 14% | 18,825 | 2,186 | (-605, 4,977) | 0.13 | 12% |  |
| DD: difference-in-differences. CI: confidence interval. UPC: Universal Product Code. Sample is balanced on stores and UPCs meaning stores and UPCs that were present in the year prior to the tax and year following the tax were included in the analysis. Model: linear DD regression with UPC fixed effects with standard errors clustered at the UPC level. Beverages with unknown taxed status or unknown beverage category are omitted. | | | | | | | | | | | |
