# Supplementary material for: Investigation of spillover effects of a sugar-sweetened beverage tax on beverage purchasing in a nearby, non-taxed area: A quasi-experimental, difference-in-differences analysis
Source: PLoS One. 2026 Feb 4;21(2):e0340577. doi: 10.1371/journal.pone.0340577 (PMC12872015; doi:10.1371/journal.pone.0340577)
Supplement: S2 Table — This table presents results from a secondary analysis that did not restrict the sample to a panel dataset. (DOCX) [file pone.0340577.s003.docx]

**S2 Table.** Difference-in-differences in mean volume sold (liters) in Seattle and King County versus comparison areas by taxed status comparing two years before to two years after the Seattle Sweetened Beverage Tax in an unbalanced sample of UPCs 2016-2019.

|  | King County excluding Seattle (KC) vs. Comparison Area | | | | | Seattle vs. Comparison Area | | | | |
| --- | --- | --- | --- | --- | --- | --- | --- | --- | --- | --- |
|  | Pre-tax mean | DD Estimate | 95% CI | *P* value | Percent change from pre-tax | Pre-tax mean | DD estimate | 95% CI | *P* value | Percent change from pre-tax |
| **Taxed Beverages** |  |  |  |  |  |  |  |  |  |  |
| Overall | 13,564 | **2,212** | **(675, 3,748)** | **0.01** | **16%** | 10,870 | **-1,555** | **(-2,442, -668)** | **<0.01** | **-14%** |
| *Beverage Type* |  |  |  |  |  |  |  |  |  |  |
| Soda | 7,949 | **3,516** | **(1,236, 5,795)** | **0.003** | **44%** | 8,027 | **-2,000** | **(-3,277, -724)** | **<0.01** | **-25%** |
| Fruit Drinks | 5,305 | 1,223 | (-2,507, 4,953) | 0.52 | 23% | 6,250 | -1,531 | (-3,755, 693) | 0.18 | -24% |
| Bottled Coffee | 6,821 | 821 | (-1,388, 3,030) | 0.47 | 12% | 4,898 | 28 | (-1,450, 1,505) | 0.97 | 1% |
| Bottled Tea | 7,914 | 1,200 | (-1,452, 3,852) | 0.38 | 15% | 6,628 | -1,006 | (-2,574, 563) | 0.21 | -15% |
| Energy Drinks | 23,888 | -1,154 | (-4,639, 2,332) | 0.52 | -5% | 13,629 | -1,183 | (-3,317, 952) | 0.28 | -9% |
| Sports Drinks | 46,972 | -3,370 | (-10,712, 3,972) | 0.37 | -7% | 33,064 | **-4,412** | **(-8,718, -107)** | **0.05** | **-13%** |
| *Beverage Size* |  |  |  |  |  |  |  |  |  |  |
| Single Serving (≤ 1 liter) | 6,144 | 682 | (-58, 1,422) | 0.07 | 11% | 5,356 | -274 | (-743, 195) | 0.25 | -5% |
| Multi-pack | 25,324 | **4,194** | **(971, 7,416)** | **0.01** | **17%** | 15,224 | **-1,856** | **(-3,541, -171)** | **0.03** | **-12%** |
| Family Size (> 1 liter) | 73,256 | 3,427 | (-3,603, 10,458) | 0.34 | 5% | 50,027 | **-5,829** | **(-10,129, -1,529)** | **0.01** | **-12%** |
| **Nontaxed Beverages** |  |  |  |  |  |  |  |  |  |  |
| Overall | 9,931 | **3,064** | **(130, 5,999)** | **0.04** | **31%** | 15,024 | 300 | (-1,375, 1,974) | 0.73 | 2% |
| *Beverage Type* |  |  |  |  |  |  |  |  |  |  |
| Diet Soda | 22,571 | 1,735 | (-2,504, 5,975) | 0.42 | 8% | 15,311 | 718 | (-2,210, 3,645) | 0.63 | 5% |
| 100% Juice/Diet Fruit Drinks | 6,244 | 269 | (-1,985, 2,524) | 0.82 | 4% | 5,767 | -403 | (-1,907, 1,102) | 0.60 | -7% |
| Milk | 32,222 | **11,159** | **(1,447, 20,871)** | **0.02** | **35%** | 22,831 | 2,582 | (-2,525, 7,689) | 0.32 | 11% |
| Bottled Coffee | 3,025 | 1,834 | (-140, 3,808) | 0.07 | 61% | 2,378 | 1,951 | (-59, 3,961) | 0.06 | 82% |
| Bottled Tea | 5,671 | 1,373 | (-1,452, 4,197) | 0.34 | 24% | 4,118 | 795 | (-647, 2,237) | 0.28 | 19% |
| Plain/Sparkling/Flav. Water | 15,613 | 1,824 | (-12,763, 16,411) | 0.81 | 12% | 23,688 | -2,187 | (-10,068, 5,693) | 0.59 | -9% |
| Diet Energy Drinks | 18,167 | 2,768 | (-955, 6,491) | 0.14 | 15% | 11,457 | 1,067 | (-1,411, 3,546) | 0.40 | 9% |
| Diet Sports Drinks | 49,263 | 2,605 | (-6,351, 11,562) | 0.57 | 5% | 32,252 | 709 | (-5,354, 6,773) | 0.82 | 2% |
| *Beverage Size* |  |  |  |  |  |  |  |  |  |  |
| Single Serving (≤ 1 liter) | 14,013 | 386 | (-218, 991) | 0.21 | 3% | 9,994 | -7 | (-396, 382) | 0.97 | 0% |
| Multi-pack | 64,462 | 4,444 | (-5,356, 14,244) | 0.37 | 7% | 49,483 | 691 | (-4,988, 6,369) | 0.81 | 1% |
| Family Size (> 1 liter) | 82,932 | 8,234 | (-998, 17,467) | 0.08 | 10% | 55,785 | -757 | (-5,900, 4,385) | 0.77 | -1% |
| DD: difference-in-differences. CI: confidence interval. UPC: Universal Product Code. | | | | | | | | | | |
| Notes: Sample is balanced on stores but not beverages (defined by the UPC). This sample allows for any UPC to enter or leave the dataset from pre- to post-tax. Analysis uses a linear DD regression model adjusted for beverage type and size with standard errors clustered at the UPC level. Beverages with unknown taxed status or unknown beverage category are omitted. | | | | | | | | | | |
