# Supplementary material for: Investigation of spillover effects of a sugar-sweetened beverage tax on beverage purchasing in a nearby, non-taxed area: A quasi-experimental, difference-in-differences analysis
Source: PLoS One. 2026 Feb 4;21(2):e0340577. doi: 10.1371/journal.pone.0340577 (PMC12872015; doi:10.1371/journal.pone.0340577)
Supplement: S1 Fig — This figure displays the coefficients of a priori event studies comparing the differences in the monthly beverage volume sold in the treated area versus comparison area prior to tax implementation. This analysis helped assess the extent to which the parallel trends assumption was reasonable in the primary differences-in-differences analysis. (DOCX) [file pone.0340577.s004.docx]

**S1 Fig.** Event study plots of the monthly mean volume sold of taxed and nontaxed beverages in King County excluding Seattle (KC) and Seattle relative to the comparison areas in the two years preceding implementation of the Seattle Sweetened Beverage Tax, 2016-2017.

1. **KC and comparison areas**


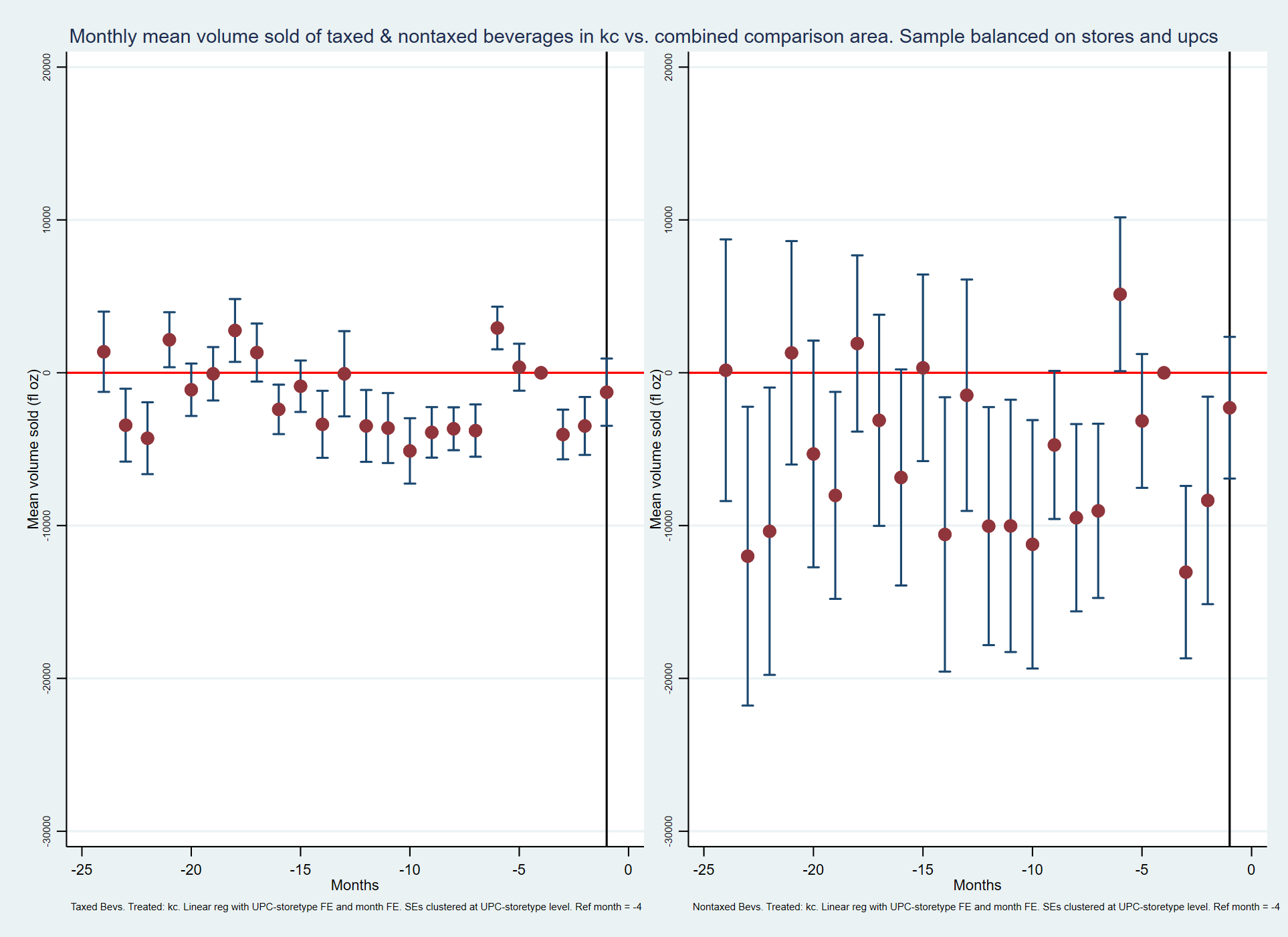


Nontaxed beverages

Taxed beverages

1. **Seattle and comparison areas**


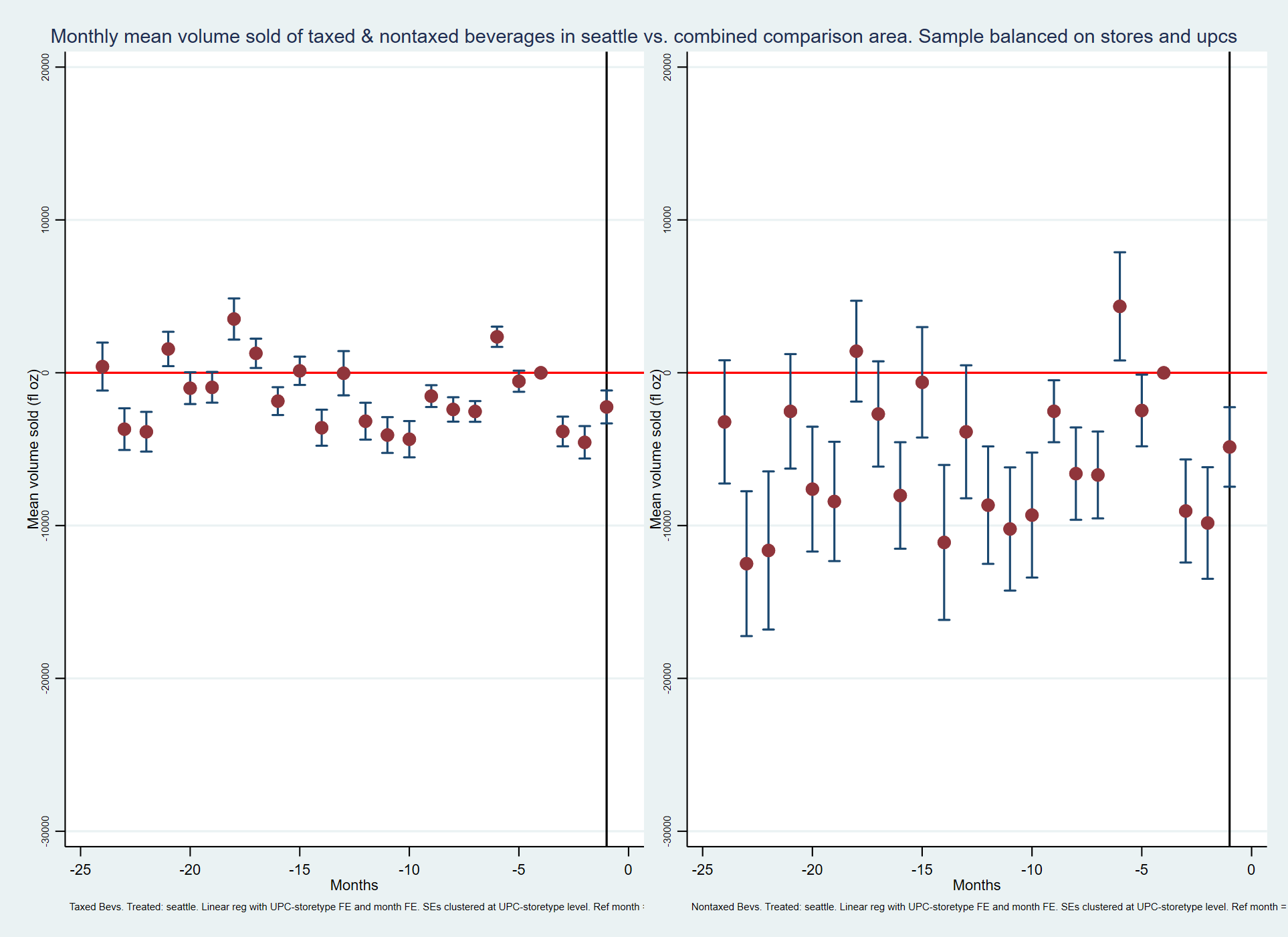


Nontaxed beverages

Taxed beverages

Note: Reference month = -4. Estimates are from a linear regression model with Universal Product Code (UPC), store type, and month fixed effects. The x axis refers to the month in which the tax was implemented: 2018, i.e., month = 0. Comparison areas: the combined area of Sacramento County, CA, and Oakland County, MI, for the KC treated area, and the combined area of Dane County, WI, and Denver County, CO, for the Seattle treated area.
