# Supplementary material for: Investigation of spillover effects of a sugar-sweetened beverage tax on beverage purchasing in a nearby, non-taxed area: A quasi-experimental, difference-in-differences analysis
Source: PLoS One. 2026 Feb 4;21(2):e0340577. doi: 10.1371/journal.pone.0340577 (PMC12872015; doi:10.1371/journal.pone.0340577)
Supplement: S2 Fig — The figure displays the coefficient results of an event study comparing the differences in annual beverage volume in the treated vs. comparison areas relative to the last year preceding tax implementation. (DOCX) [file pone.0340577.s005.docx]

**S2 Fig.** Differences in differences in annual mean volume sold of taxed and nontaxed beverages in King County excluding Seattle (KC) and Seattle relative to the comparison areas from two years before and after implementation of the Seattle Sweetened Beverage Tax, 2016-2019.

**A) KC and comparison areas**


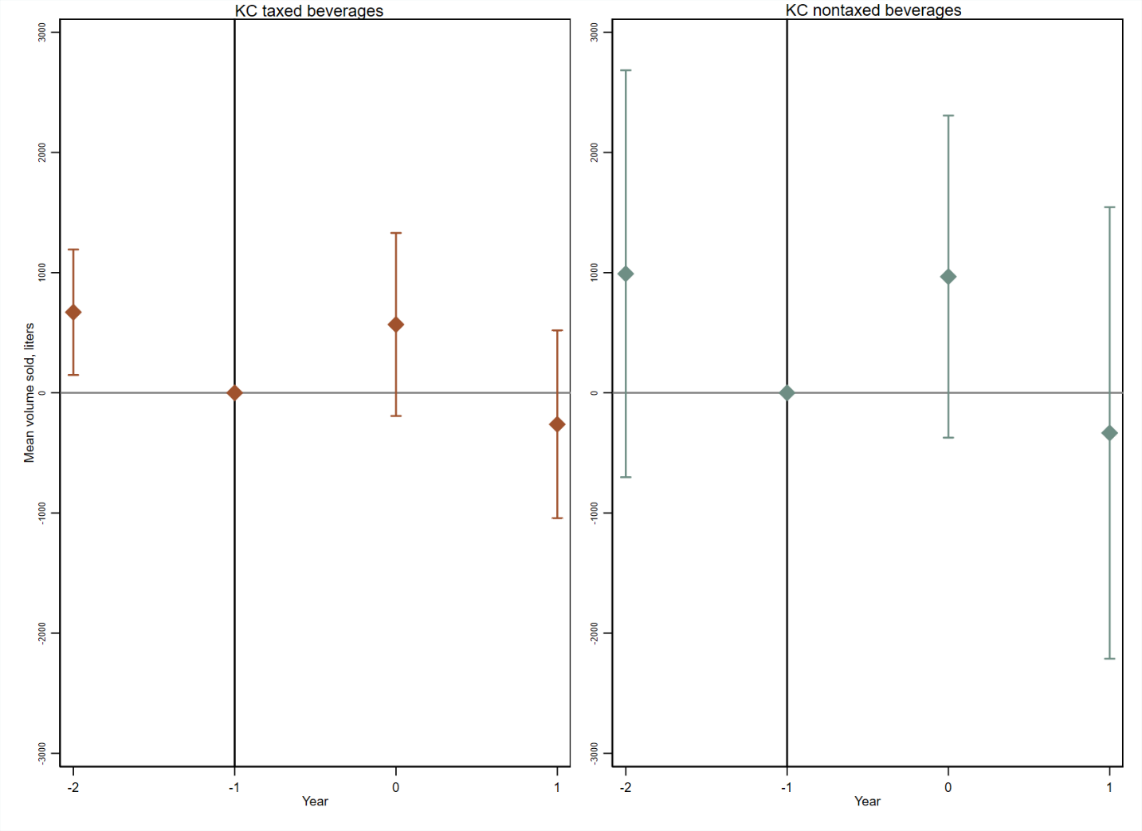


**B) Seattle and comparison areas**


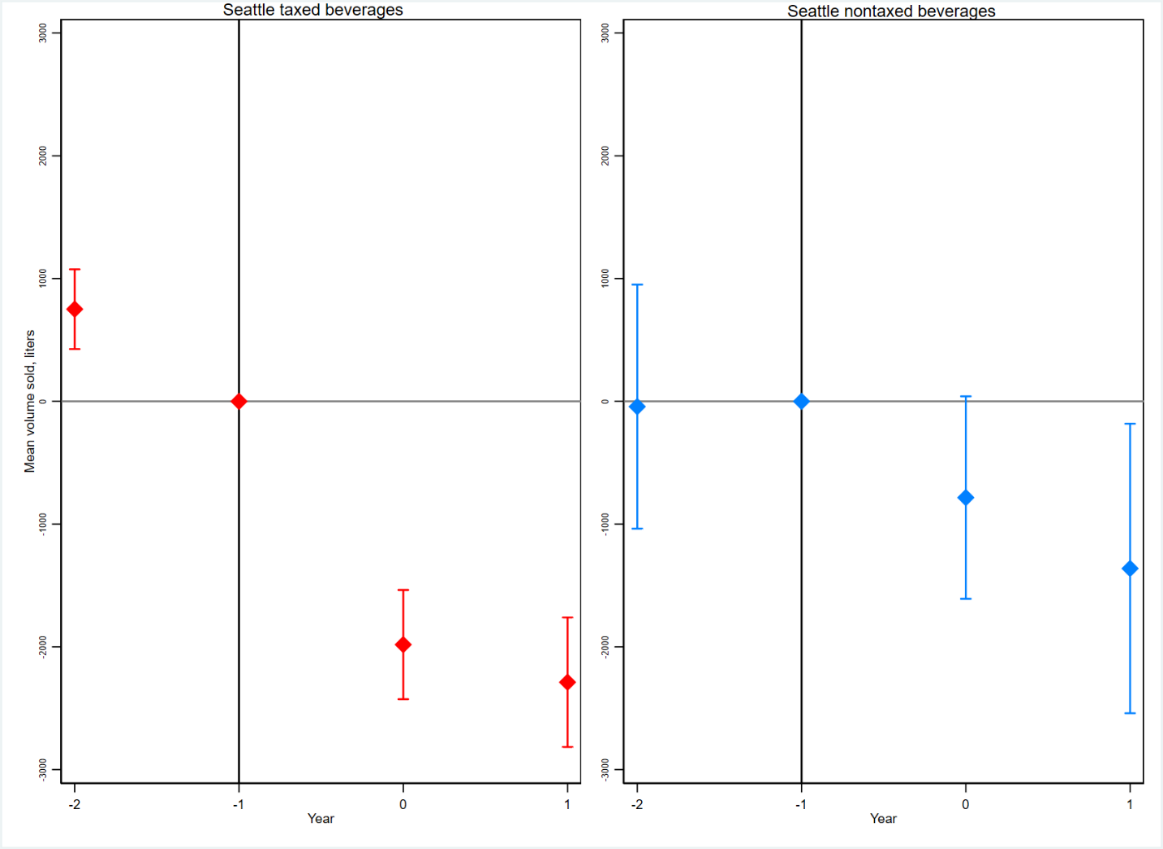


Note: Estimates are from a linear regression model with Universal Product Code (UPC) fixed effects and year fixed effects. Standard errors are clustered at the UPC level. The x axis refers to the year in which the SSB tax was implemented: 2018, i.e., Year = 0. Comparison areas: the combined area of Sacramento County, CA, and Oakland County, MI, for the KC treated area, and the combined area of Dane County, WI, and Denver County, CO, for the Seattle treated area.
